# Navigating the Pharma 4.0 revolution: how AI-enabled applications and predictive modelling shaped pharmaceutical development in the post-pandemic era: a systematic review

**DOI:** 10.3389/frai.2026.1976101

**Published:** 2026-10-30

**Authors:** Xin Yi Chang, Clarence Teo, Cheong Hian Goh

**Affiliations:** 1 Department of Pharmacy & Pharmaceutical Sciences, National University of Singapore, Singapore, Singapore; 2 Health Sciences Authority, Singapore, Singapore

**Keywords:** artificial intelligence, COVID-19 pandemic, drug development, Pharma 4.0, pharmaceutical industry

## Abstract

Pharma 4.0 is touted to make processes more efficient and resilient to unforeseen circumstances, boosting productivity, speed and accuracy of pharmaceutical systems. Besides operational stability, these technological implementations could potentially improve clinical outcomes. AI-enabled Pharma 4.0 applications and predictive modelling are utilised in pharmaceutical development for the discovery of new compounds that could potentially be used as drugs for COVID-19. This systematic review examined how contemporary Pharma 4.0 technologies have impacted pharmaceutical development in the post COVID-19 pandemic. A systematic review following the “Preferred Reporting Items for Systematic Review and Meta-Analyses” (PRISMA) method was carried out on scientific peer-reviewed literature, covering the period from 2023 to 2025. From the PubMed and Scopus databases, 593 articles were identified and 57 were included for further analysis. Performance metrics describing the reported predictive models used in research areas were identified and descriptive statistics were used to present the findings. We found that predictive models of Pharma 4.0 technologies were predominantly concentrated in early-stage drug discovery and computational research, with reported benefits of more efficient and accurate processes. Our current findings on the nonclinical performance metrics observed good use cases for drug discovery, but limited insights on the clinical applications that these recent technological developments had on the pharmacological effects. These technologies enhanced complex processes, facilitated predictive models to optimise product development in the early phase of the product life cycle.

## 1 Introduction

Pharma 4.0 is often touted to make processes more efficient and resilient to unforeseen circumstances, boosting productivity, speed and accuracy of pharmaceutical systems. The technologies in Pharma 4.0 are rapidly evolving, with expanding scope in the components of applications and are becoming essential features in the pharmaceutical processes (Mohan et al., 2024).

Figure 1 illustrates the evolution of Industry 1.0 to 4.0 and outlines the emergence and applications of Pharma 4.0 within the Industry 4.0 framework. Since the late 18th century, manufacturing processes have gone through immense evolution to adapt to the growing needs of society. This transition is named the industrial revolution, which describes the period of scientific and technological development that changed the mainstream method of production to more efficient, productive and connected means. The First Industrial Revolution (Industry 1.0) in the late 18th century introduced mechanized production powered by water and steam, shifting production away from reliance on manual labour. One hundred years later, Industry 2.0 introduced electricity, assembly lines, and standardised processes, enabling efficient mass production. This was followed by Industry 3.0 in the 1970s, signalling the start of the digital age, which incorporated electronics, computers, information technology, and automation into industrial processes. Building upon this digital foundation, Industry 4.0 integrates intelligent and interconnected technologies, including artificial intelligence (AI), machine learning (ML), the Internet of Things (IoT), big-data analytics, robotics, and cloud computing, to create increasingly autonomous and data-driven systems (Yin et al., 2017; Klingenberg et al., 2022). These technological transformations have extended into the pharmaceutical industry, giving rise to Pharma 4.0, where digital technologies are integrated across the pharmaceutical product life cycle (Sharma et al., 2023; ISPE, 2021; Kappeler et al., 2021).

**Figure 1:**
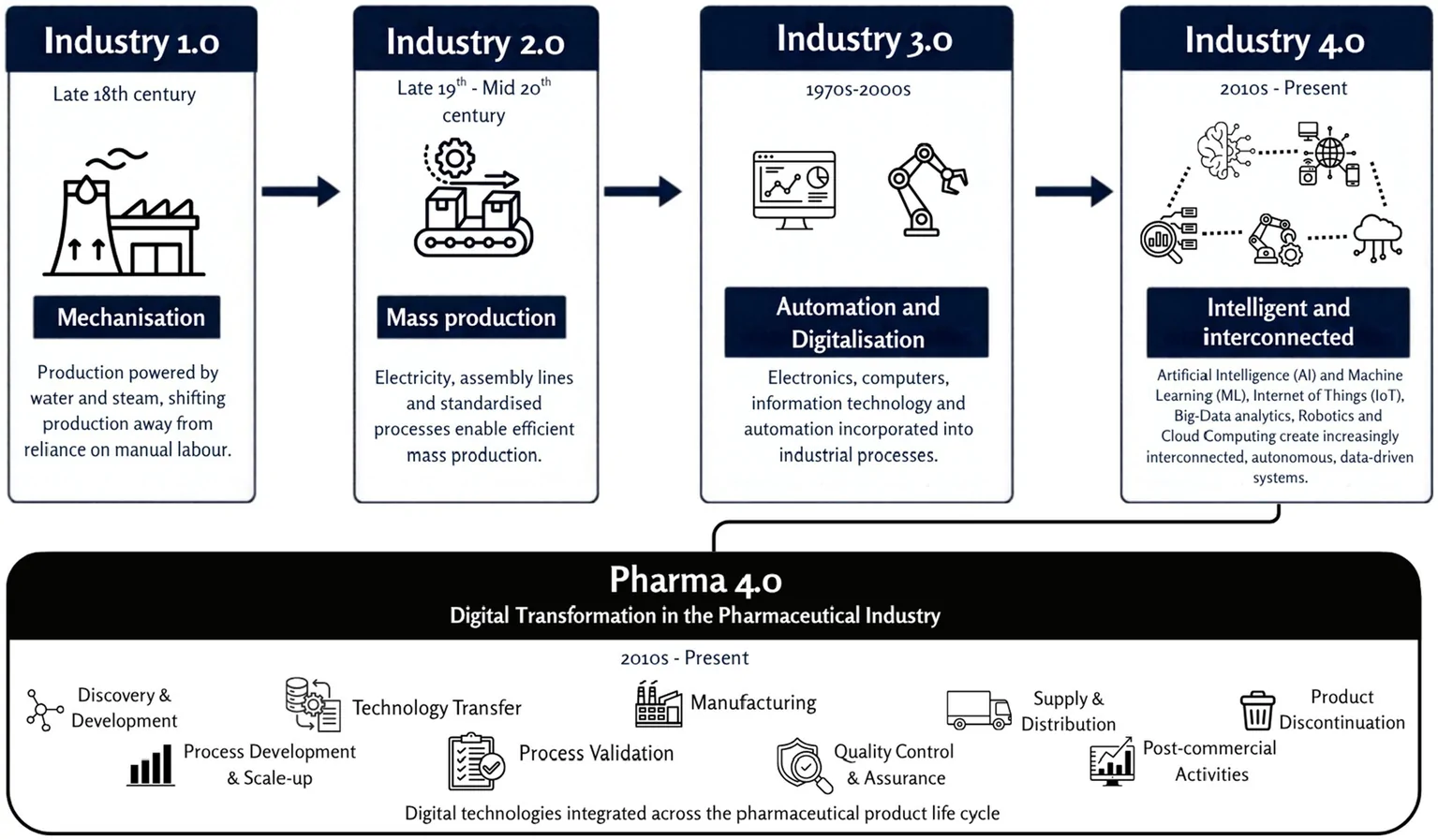
Evolution of industry 1.0 to 4.0 and Pharma 4.0.

Figure 2 depicts the transition of the pharmaceutical industry from conventional pre-COVID-19 practices towards an increasingly digitalised Pharma 4.0 ecosystem. Prior to the pandemic, pharmaceutical operations relied heavily on manual labour and heterogeneous practices. Data was fragmented in silos, and supply chains were found to have many vulnerabilities. These gaps in workflows were exposed and amplified by the pandemic (Cundell et al., 2020). Restrictions on border movement of goods and services led to the interference of supply chain services, and mandated social isolation forced workers to work remotely (Farrell et al., 2020). The disruption of pharmaceutical workflows, particularly in the manufacturing and distribution sector, further amplified the existing shortages of critical medications and equipment (Al Khzem and Wali, 2025) essential for health conditions and have serious implications on patients’ survival (Inuwa et al., 2022). To restore functions back to baseline and cope with the pandemic, companies urgently needed to optimise their workflows to adapt to the new circumstances.

**Figure 2:**
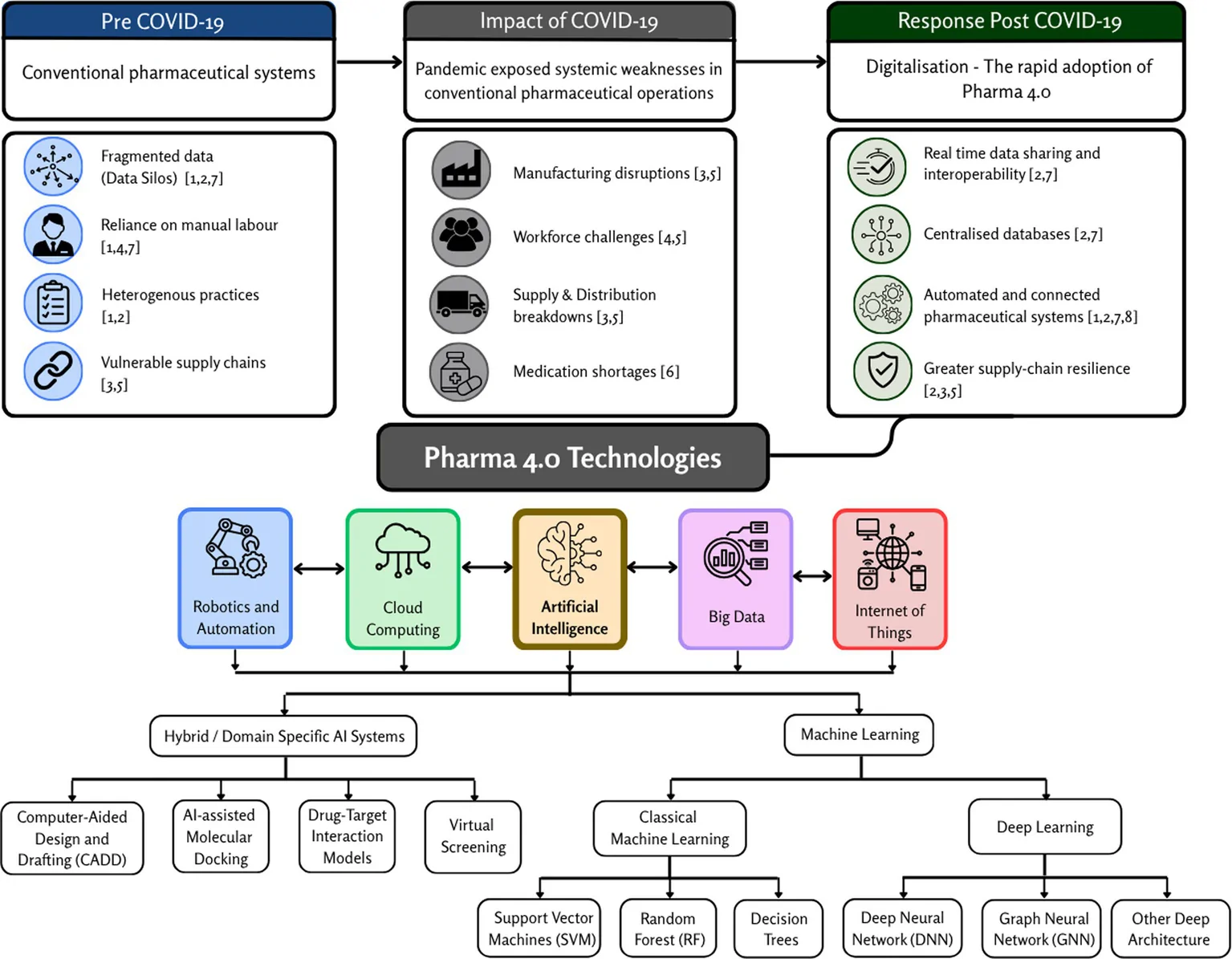
Evolution and components of Pharma 4.0, and artificial intelligence (AI) specific technologies. [1] - Mohan et al., 2024; [2] - Prescott et al., 2022; [3] - Cundell et al., 2020; [4] - Farrell et al., 2020; [5] - Al Khzem and Wali, 2025; [6] - Inuwa et al., 2022; [7] - Shinde et al., 2023; [8] - Selvaraj et al., 2021.

The response accelerated the transition towards digitalisation in the post-COVID-19 environment, through the adoption and integration of Pharma 4.0 technologies across the pharmaceutical life cycle. Figure 2 identifies the five interconnected pillars of industry 4.0: robotics and automation, cloud computing, artificial intelligence (AI), big data, and the Internet of Things (IoT). Their integration into pharmaceutical workflows provides real-time data sharing and interoperability, and centralisation and integration of information and practices. This forms an interconnected ecosystem of data networks, helping automate and streamline previously inefficient or human reliant processes (Socal et al., 2021). Ultimately, this strengthened supply chain has numerous benefits throughout the product life cycle. These technological advancements are strengthening healthcare infrastructure, with the potential to improve clinical outcomes and enhance the resilience of global public health systems (Prescott et al., 2022).

AI further consists of hybrid and machine learning approaches. Several domain specific AI systems include computer-aided drug design and drafting (CADD), AI-assisted molecular docking, drug–target interaction modelling, and virtual screening. Machine learning is further categorised into classical approaches, including support vector machines, random forests, and decision trees, and deep-learning approaches, including Deep Neural Networks (DNN) and Graph Neural Networks (GNN). These AI capabilities can complement traditional computational approaches in the early drug discovery process, and strengthen connectivity between computational stages into a data driven discovery pipeline (Selvaraj et al., 2021). AI drug predictive models use machine learning and deep learning to forecast molecular properties, pharmacokinetics, and biological toxicity (Niazi, 2025). These predictive models can improve how researchers prioritise an enormous chemical and biological search space, potentially reducing redundant experiments and computational calculations while identifying patterns that conventional approaches may overlook (Shinde et al., 2023). The digitalisation of drug discovery is expected to bring tangible benefits, including faster lead discovery and lower cost (Niazi, 2025).

The unprecedented situation has led to a growing scientific interest in understanding the progress of Pharma 4.0 technologies in the pharmaceutical development in the post-pandemic era. We found that secondary literature about Pharma 4.0 technologies are often descriptive, following the authors’ opinions specific to their pharmaceutical companies’ workflows and the improvements made (Prescott et al., 2022; Selvaraj et al., 2021; Niazi, 2025). In this systematic review, we aim to investigate further how the adoption of Pharma 4.0 technologies, in particular AI Predictive Models, affected early-stage drug discovery. Our study aimed to provide current insights made by the contemporary Pharma 4.0 in pharmaceutical development.

The objective of this review was to identify trends between the performance metrics used in predictive model studies, the type of AI technology used, and its area of research (Appendix A–C). It centred around the research and development of predictive models specialised in compound screening and drug discovery studies.

## 2 Methodology

This study employed the Systematic Literature Review methodology to understand the use of Pharma 4.0 technologies in different stages of the product life cycle after the pandemic and the performance metrics used to evaluate them. The PICO framework has been widely applied in systematic literature reviews (Fitzgerald et al., 2025) to formulate research questions and guide the literature selection process. Accordingly, the PICO framework was adopted in this study as follows:

P (Population): Pharmaceutical companies

I (Intervention): Adoption of Pharma 4.0

C (Comparator): Pre-pandemic vs. post-pandemic practices

O (Outcome): Effect on product life cycle management

Hence, this Systematic Literature Review was conducted to address the following research question: How has the adoption of Pharma 4.0 affected the product life cycle management after the pandemic?

The following research objectives were to study:

1. extent of implementation of AI-enabled applications and predictive modelling in Pharma 4.0
2. advantages and disadvantages of AI-enabled applications and predictive modelling in Pharma 4.0
3. how AI-enabled applications and predictive modelling in Pharma 4.0 have affected the integration between the stages of the product life cycle

### 2.1 Database selection

Two major electronic databases, namely PubMed and Scopus, were selected as sources to look for relevant documents for the study. These two databases are commonly used by researchers and comprise literature such as peer reviewed articles and papers from the biomedical field. The literature search was conducted from 25th August 2025 to 5th February 2026.

### 2.2 Keyword selection

The keywords selected for this study covered 3 main areas of the research question:

Pharma 4.0 technology, pharmaceutical industry and COVID-19 pandemic (Table 1).

**Table 1:** Keywords used in search strategy.

COVID-19	Pharma 4.0	Pharmaceutical industry
SARS-CoV-2, COVID-19, coronavirus infections	Industry 4.0, artificial intelligence, machine learning, internet of things, automation	Pharmaceutical research, development, manufacturing

We defined the post-pandemic period for the inclusion criteria to be from 2023 to 2025, and excluded pandemic-era studies from 2020 to 2022. This took into consideration the World Health Organization’s official declaration on 5th May 2023, that ended COVID-19’s Public Health Emergency of International Concern status (Roknuzzaman et al., 2024). This also highlights the end of an unprecedented situation in which AI/ML adoption was accelerated by the urgent need for COVID-19 therapeutics. By focusing on post-pandemic studies, this study provided a more representative assessment of the sustained integration of AI/ML into early-stage drug discovery practices of the post-pandemic Pharma 4.0 context.

### 2.3 Articles selection

We followed the Preferred reporting Items for Systematic Review and Meta-Analyses (PRISMA) 2020 framework (PRISMA, 2020) and applied the 4 steps: identification, screening, eligibility and final inclusion when selecting the articles (see Figure 3). Articles were screened, using the inclusion and exclusion criteria, and those that were relevant to the study were included for further analysis.

**Figure 3:**
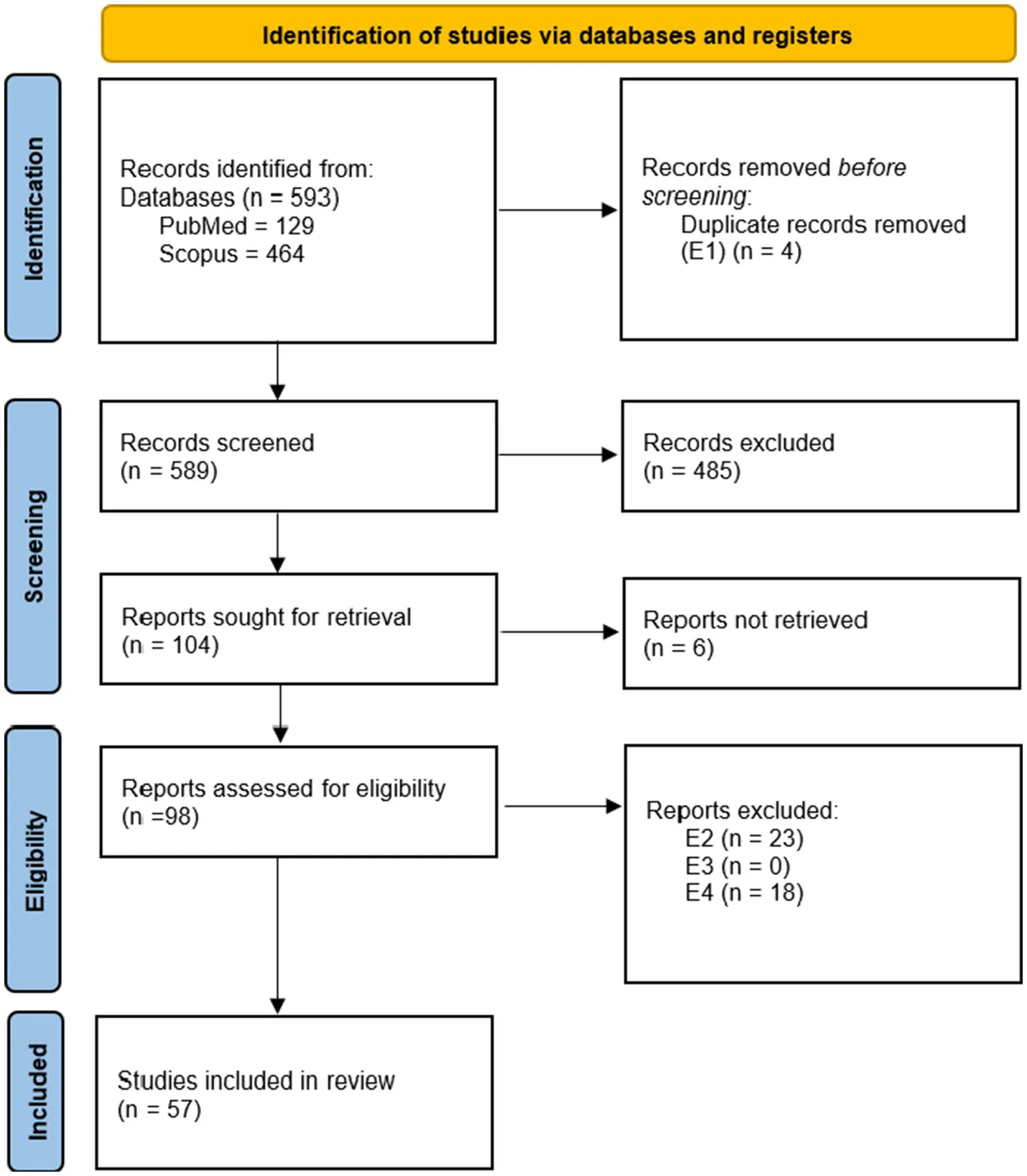
Preferred Reporting Items for Systematic reviews and Meta-Analyses (PRISMA) 2020.

#### 2.3.1 Identification

The search strategy (Appendix D) was created using keywords (Table 1) and was applied to both PubMed and Scopus to identify the list of 593 articles. 4 duplicates were then removed from this initial list to obtain a total of 589 articles.

#### 2.3.2 Screening

The initial list of articles was then screened by only their title and abstract. During the screening process, each researcher conducted the screening independently. Inter-reviewer agreement was assessed prior to consensus resolution. Of the 589 records independently assessed by two reviewers, agreement was reached on 566 records (96.1%), comprising 95 records selected for inclusion and 471 records excluded. The reviewers disagreed on 23 records (3.9%). The Cohen’s kappa demonstrated a high level of inter-reviewer agreement (*κ* = 0.87). All disagreements were subsequently resolved through discussion and consensus, resulting in 104 records being included and considered for full text retrieval while 485 records being excluded. However, 6 of the articles did not have full text available for retrieval, thus the number of reports assessed for eligibility was 98. When consensus could not be achieved, a third reviewer was consulted to provide an independent assessment and facilitate resolution of the disagreement.

#### 2.3.3 Eligibility

Full text screening was then conducted separately by each of the researchers. Articles that were related to the research questions and objectives were considered to be eligible. 41 articles were excluded according to the respective exclusion criteria (Table 2).

**Table 2:** Inclusion and exclusion criteria used during screening of articles.

Inclusion criteria	Exclusion criteria
- Studies published from 2023 to 2025 - Include peer-reviewed articles and conference papers only - Able to access full text articles - Answers any of the research objectives mentioned above - Monitoring parameters/performance metrics used	- E1: Exclude duplicates - E2: Exclude reviews, surveys, book chapters - E3: Studies not written in English language - E4: No relevance to research question and objectives

#### 2.3.4 Final inclusion

After excluding 41 articles, the remaining 57 articles were included in the review which comprises articles related to the use of Pharma 4.0 technologies in pharmaceutical development. The results of these studies are analysed and discussed in the following sections. The data extraction process was conducted separately by two researchers using a coding manual (Appendix E) to ensure consistency and minimize potential bias when identifying keywords used in the conclusion. Data such as the performance metrics used, key findings of the paper as well as keywords used to describe the application of AI and ML were extracted. Any discrepancies or disagreements identified during the data extraction process were resolved through discussion and comparison of the extracted information against the original articles. The researchers reviewed the relevant sections of each article and reached a consensus based on the information reported. Similarly, when consensus could not be reached between the two reviewers, a third independent reviewer was consulted to determine the final decision. This approach enhances the accuracy, reliability, and reproducibility of the extracted data.

### 2.4 Risk of bias

The Prediction model study Risk of Bias Assessment Tool (PROBAST) tool (PROBAST, 2020), which evaluates prediction models, was used to evaluate the risk of bias of the included articles (Appendix F). Although PROBAST was originally developed for diagnostic and prognostic prediction models, it was considered the most applicable established risk of bias framework for this review as the included studies predominantly involved the development or evaluation of predictive models. The PROBAST domains relating to data sources, predictors, outcomes, and statistical analysis capture key methodological concerns relevant to AI-based drug-discovery models. Alternative established risk of bias tools are predominantly designed for other study types, such as randomized trials, non-randomized intervention studies, or diagnostic accuracy studies, and were therefore not suitable for the model-development focus of the included literature.

These papers are in early-stage drug discovery and often lack external validation, hence the majority of the articles were assessed to be of high risk of bias. This reflects methodological limitations inherent to early-stage drug discovery and that the articles are more suitable for hypothesis generation than in clinical application.

## 3 Results

Table 3 illustrates the performance metrics found in the 57 articles that were included for the systematic review, which consisted of predictive models used in early stages of the drug development. The profile of predictive models in these articles was characterised by Drug Candidate Metrics, Computational Evaluation Metrics, and AI/ML Model Evaluation Metrics (Appendix B).

**Table 3:** Distribution of the performance metrics described in the 57 articles^#^.

Drug candidate evaluation metrics evaluate drug candidate properties		Computational evaluation metrics evaluate drug-target relationship prediction performance		AI/ML model evaluation metrics evaluate machine learning model performance			
				Classification and curve metrics		Regression metrics	
IC50/pIC50	9	RMSD	6	AUC-ROC/AUROC/AUC	19	RMSE	10
EC50	2	Docking score	3	Sensitivity/Recall /TPR	17	R2	8
QED	2	RMSF	3	Accuracy	16	Pearson’s coefficient	5
pLogP	2	Binding Energy	2	Precision	13	MSE	4
				F1 score	10	MAE	4
				MCC	9		
				Specificity	4		
				AUPRC	3		
				BACC	1		

^#^Each performance metric described in the article is counted and presented as an aggregated number for each category in the table. Most of the articles reviewed mention one or more performance metrics in their methodology.

As such, the total count of each category does not add up to 57.

Firstly, the drug candidate evaluation metrics would measure the biological, chemical or structural properties of the candidate molecules mentioned in their respective article. These experimental data are provided by public chemical and biological databases, such as PubChem and BindingDB, and converted to training and validation datasets for predictive models to analyse chemical structures and predict drug-related outcomes.

Secondly, computational evaluation metrics were used to computationally evaluate predicted interactions between candidate molecules and biological targets. These measures characterise predicted molecular interactions and structural behaviour between drug candidate and target. The results obtained are used for filtering out viable drug candidates from an enormous chemical search space before experimental validation.

Thirdly, the AI/ML model evaluation metrics would evaluate different aspects of the predictive model’s performance by assessing the accuracy of the outcomes generated from raw data. Classification metrics evaluate models in predicting discrete outcomes, such as accuracy of prediction or activity of molecule. Regression metrics assess and quantify how closely the predicted continuous values, such as binding affinity and IC50, compare with experimentally observed values from the chemical databases. These 2 subcategories provide an overall assessment of the model’s predictive capability and reliability, which are commonly used by researchers to compare the predictive performance between various predictive models.

Across the 57 included articles, a total of 152 individual metric mentions were identified. Percentages were calculated using the total number of metric mentions (*n* = 152) as the denominator, rather than the total number of included articles (*n* = 57), as individual articles could report more than one evaluation metric. AI/ML model evaluation metrics accounted for 80.9% (123/152) of all metric mentions, comprising classification metrics (60.5%, 92/152) and regression metrics (20.4%, 31/152). Drug candidate evaluation metrics accounted for 9.9% (15/152) of mentions, while computational evaluation metrics accounted for 9.2% (14/152). At the individual-metric level, AUC-ROC/AUROC/AUC was the most frequently reported classification metric (12.5%, 19/152), RMSE was the most frequently reported regression metric (6.6%,10/152), IC50/pIC50 was the most frequently reported drug candidate evaluation metric (5.9%, 9/152), and RMSD was the most frequently reported computational molecular evaluation metric (3.9%, 6/152). The percentage distribution of the performance metrics found in the 57 articles extracted is shown in Figure 4.

**Figure 4:**
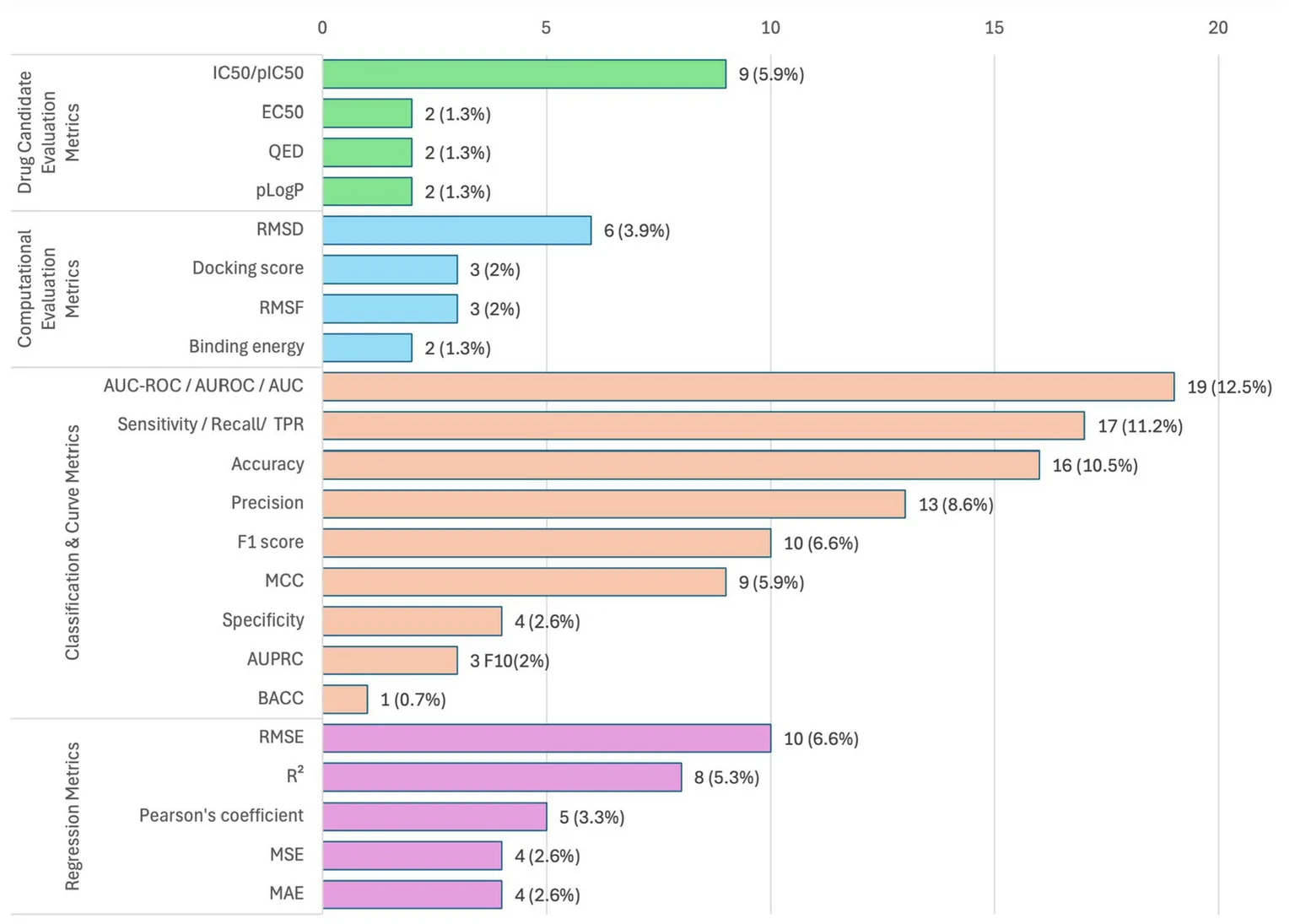
Distribution of performance metrics across included studies. # Percentages represent the proportion of total metric mentions (*n* = 152), calculated with a formula $\text{Percentage of metric mentions}\;(\%)=\frac{\text{Number of mentions of the metric or category}}{\text{Total metric mentions}\;(n=152)}\times 100.$

As presented in Table 4, the 57 articles reviewed in our study reported applications of predictive models to different research areas. We grouped the articles according to the Pharma 4.0 technology and the research area, as well as the performance metrics described in these studies. The articles were categorised by the specific technology used (Deep Neural Network, Graph Neural Network), otherwise it would be categorised under the broader terms (Deep Learning, Machine Learning or Artificial Intelligence). The key findings are also listed in the form of the data presented in each study.

**Table 4:** Summary of reviewed literature.

No.	Title	Author	Research area	Metrics	Key findings	Conclusion
Artificial intelligence/machine learning						
1	Open science discovery of potent noncovalent SARS-CoV-2 main protease inhibitors (Boby et al., 2023)	Boby et al.	Drug discovery/design	IC50, EC50	IC50^b^: 37 nM EC50^b^: 64 nM	Effective, quick
2	AI-accelerated protein-ligand docking for SARS-CoV-2 is 100-fold faster with no significant change in detection (Clyde et al., 2023)	Clyde et al.	Docking studies	MAE, R^2	MAE^b^: 0.54–0.74 R^2^a^: 0.71 to 0.85	Quick, effective
3	Artificial intelligence-based HDX (AI-HDX) prediction reveals fundamental characteristics to protein dynamics: mechanisms on SARS-CoV-2 immune escape (Yu et al., 2023)	Yu et al.	Computational Prediction	RMSE	RMSE^b^: 0.25	Universally applicable
4	Leveraging machine learning to streamline the development of liposomal drug delivery systems (Eugster et al., 2024)	Eugster et al.	Computational Prediction	AUC, AUROC, precision, accuracy, R^2	R^2^a^: 0.74 ± 0.08 AUC^a^: 0.98 ± 0.01 Accuracy^a^: 0.5–1.0	Universally applicable, effective, reliable, robust, accurate
5	Repurposing of drugs for combined treatment of COVID-19 cytokine storm using machine learning (Gantla et al., 2023)	Gantla et al.	Computational Prediction	ACC, TPR, FPR, Precision, MCC, AUROC, PRC area, binding energy	ACC^a^: 91.5–98.55% TPR^a^: 0.915–0.985 FPR: 0.015–0.085 Precision^a^: 0.915–0.985 MCC^a^: 0.830–0.970 AUROC^a^: 0.978–0.999 PRC area: 0.979–0.999 Average binding affinity^a^: −6.40 to −8.37 kcal/mol	Effective, accurate
6	FragOPT: an ML-driven computational workflow for rational fragments optimization toward lead compounds (Wu et al., 2025)	Wu et al.	Drug discovery/design	Accuracy, Precision, Recall, F1 score, MCC, AUC	Accuracy^a^: 0.878 Precision^a^: 0.893 Recall^a^: 0.971 F score^a^: 0.912 MCC^a^: 0.718 AUC^a^: 0.944	Effective
7	*De Novo* design of anti-COVID drugs using machine learning-based equivariant diffusion model targeting the spike protein (Niranjan et al., 2023)	Niranjan et al.	Drug discovery/design	RMSD, RMSF	RMSD^b^: 5.8–7.2 angstroms	Applicable for large dataset
8	Accelerating reliable multiscale quantum refinement of protein–drug systems enabled by machine learning (Yan et al., 2024)	Yan et al.	Drug discovery/design	core-hours	Core hours^a^: 6.7–31.5	Effective, cost saving
Virtual screening						
9	Virtual and *in vitro* screening of natural products identifies indole and benzene derivatives as inhibitors of SARS-CoV-2 main protease (Mpro) (Ang et al., 2023)	Ang et al.	Virtual Screening	AUROC	AUROC^a^: 0.92	Quick, accurate
10	Accelerating molecular docking using machine learning methods (Bande and Baday, 2024)	Bande and Baday	Docking studies	F1 score, accuracy, AUROC, RMSE	F1 score^a^: 0.52 Accuracy^a^: 0.85 RMSE^b^: 1.9–2.3	Quick, cost saving
11	Development of machine learning regression model for COVID-19 drug target prediction (Zamitalo et al., 2023)	Zamitalo et al.	Docking studies	MSE, MAE, R^2, docking score	MSE^b^: 0.627 MAE^b^: 0.285 R^2^a^: 0.690	Quick, efficient
12	Effective surrogate models for docking scores prediction of candidate drug molecules on SARS-CoV-2 protein targets (Shi et al., 2023)	Shi et al.	Docking studies	R^2, MAE, RMSE, MAPE	R^2^a^: 0.88 MAE^b^: 0.41, 0.32 RMSE^b^: 0.47	Effective, quick, robust, accurate, universally applicable
13	Artificial intelligence-driven discovery of pyrazolo[1,5- a]pyrimidine derivatives as novel phosphodiesterase 4 inhibitors for treating idiopathic pulmonary fibrosis (Li et al., 2025)	Li et al.	Drug discovery/design	IC50	IC50^b^: 2.7 nM	Efficient
14	Applications of machine learning approaches for the discovery of SARS-CoV-2 PLpro INHIBITORS (Pal et al., 2025)	Pal et al.	Drug discovery/design	IC50, efficacy, AUC	IC50^b^: 1750–36,000 nM Efficacy^a^: >30% Overall AUC-ROC value^a^: >0.75	Effective, accurate
15	Modeling structure–activity relationships with machine learning to identify GSK3-targeted small molecules as potential COVID-19 therapeutics (Pirzada et al., 2023)	Pirzada et al.	Drug discovery/design	RMSD, RMSF, IC50, binding energy	Binding energy^a^: 8681.320 kcal//mol	Widely used
16	D2Screen: embedding pretrained representation learning model and molecular docking for virtual screening (Qian et al., 2025)	Qian et al.	Drug discovery/design	BedROC, EF1% (unique system) IC50	BedROC: >0.7 EF1%: >10 IC50^b^: 5900 nM	Effective, require little input, universally applicable, robust
Random forest model						
17	Machine learning approaches for COVID-19 classification and potential novel therapeutic targets (Elia and Duan, 2025)	Elia and Duan	Computational Prediction	accuracy, AUC, specificity, sensitivity	Accuracy^a^: 92.31% AUC^a^: 0.93	Effective, accurate, less complex
18	Developing a SARS-CoV-2 main protease binding prediction random forest model for drug repurposing for COVID-19 treatment (Liu et al., 2023)	Liu et al.	Computational Prediction	accuracy, sensitivity, specificity, MCC	Accuracy^a^: 0.514 Sensitivity^a^: 0.432 Specificity^a^: 0.723 MCC^a^: 0.143	Effective, accurate, specific, sensitive
Support vector machine						
19	Proteomic data and structure analysis combined reveal interplay of structural rigidity and flexibility on selectivity of cysteine cathepsins (Tušar et al., 2023)	Tušar et al.	SAR	TPR, accuracy range	TPR^a^: 0.802–0.848 Accuracy^a^: 80–91%	Effective, accurate
20	Quantitative structure–activity relationship study of cathepsin L inhibitors as SARS-CoV-2 therapeutics using enhanced SVR with multiple kernel function and PSO (Li S. et al., 2025)	Li et al.	Drug discovery/design	R^2, RMSE	R^2^a^: 0.9676 RMSE^b^: 0.0834	Effective
Deep Learning						
21	Integrating physics-based simulations with data-driven deep learning represents a robust strategy for developing inhibitors targeting the main protease (Yang et al., 2025)	Yang et al.	Computational Prediction	MSE, Pearson’s correlation	MSE^b^: 1.11–1.63 Pearson coefficient^a^: 0.71–0.81	Effective
22	F-CPI: a multimodal deep learning approach for predicting compound bioactivity changes induced by fluorine substitution (Zhang et al., 2024)	Zhang et al.	Computational Prediction	accuracy, precision, recall, IC50	Accuracy^a^: 89.33 Precision^a^: 66.52 Recall^a^: 43.15	Effective, applicable for large dataset
23	A deep learning framework for predicting molecular property based on multi-type features fusion (Ma and Lei, 2024)	Ma and Lei	Computational Prediction	AUC, RMSE	AUC^a^: 0.931 RMSE^b^: 0.849	Accurate, effective
24	A robust drug–target interaction prediction framework with capsule network and transfer learning (Huang et al., 2023)	Huang et al.	Computational Prediction	F1 score, accuracy, AUC, AUPR	Accuracy^a^: 0.87–0.941 F1^a^: 0.862–0.938 AUC^a^: 0.935–0.982 AUPR^a^: 0.944–0.983	Accurate, large scale, universally applicable, robust
25	How deep learning in antiviral molecular profiling identified anti-SARS-CoV-2 Inhibitors (Ali et al., 2023)	Ali et al.	Computational Prediction	EC50, AUROC	EC50^b^: 1–7 μM AUROC^a^: 0.85	Effective
26	AMMVF-DTI: a novel model predicting drug–target interactions based on attention mechanism and multi-view fusion (Wang et al., 2023)	Wang et al.	Computational Prediction	AUC, precision, recall	AUC^a^: 0.986 Precision^a^: 0.976 Recall^a^: 0.938	Effective, accurate, robust
27	Protein-ligand binding affinity prediction using multi-instance learning with docking structures (Kim et al., 2025)	Kim et al.	Computational Prediction	RMSE, MAE, R^2, pearson’s coefficient	RMSE^b^: 1.242 MAE^b^: 0.962 R^2: 0.844 Pearson^a^: 0.844	Universally applicable
28	Chem2Side: a deep learning model with ensemble augmentation (Conventional + Pix2Pix) for COVID-19 drug side-effects prediction from chemical images (Arshed et al., 2023)	Arshed et al.	Computational Prediction	Precision, recall, accuracy, F1 score	Accuracy^a^: 0.72 Precision^a^: 0.79 Recall^a^: 0.72 F1 score^a^: 0.73	Quick, effective
29	Improving protein–ligand interaction modeling with cryo-EM Data, templates, and deep learning in 2021 ligand model challenge (Giri and Cheng, 2023)	Giri and Cheng	Computational prediction	Q-score, Z-score	Q-score: 44	Quick
30	Broad-spectrum coronavirus inhibitors discovered by modeling viral fusion dynamics (Reilly et al., 2025)	Reilly et al.	Drug discovery/design	IC50, Cmax, Tmax, AUC	IC50^b^: 70 nM Cmax^a^ = 1,757 ng/mL Tmax^a^ = 13 h AUC ^a^= 33,287 h*ng/ml	Quick, large scale, effective,
31	MVCL-DTI: predicting drug-target interactions using a multiview contrastive learning model on a heterogeneous graph (Zhang et al., 2025)	Zhang et al.	Drug discovery/design	AUROC, AUPRC	AUROC^a^: 0.9677, AUPR^a^: 0.9024	Robust
32	DeepMPF: deep learning framework for predicting drug-target interactions based on multi-modal representation with meta-path semantic analysis (Ren et al., 2023)	Ren et al.	Drug discovery/design	AUC, accuracy, sensitivity, precision, F1 score, MCC	Accuracy^a^: 0.75–0.9057 AUC^a^: 0.82–0.96 Precision^a^: 0.80–0.93 Sensitivity^a^: 0.65–0.92 F1 score^a^: 0.72–0.92 MCC^a^: 0.51--0.86	Effective
33	Advancing drug–target interaction prediction: a comprehensive graph-based approach integrating knowledge graph embedding and ProtBert pretraining (Djeddi et al., 2023)	Djeddi et al.	Drug discovery/design	AUC, AUPR, ACC, MCC, F1	AUPR^a^: 0.9% higher AUC^a^: 0.1% higher MCC^a^: 1.02% higher F1 score^a^: 0.92% higher	Effective
34	Unsupervised deep learning for molecular dynamics simulations: a novel analysis of protein-ligand interactions in SARS-CoV-2 Mpro (Mustali et al., 2023)	Mustali et al.	Drug discovery/design	pIC50, IC50, pearson’s coefficient	IC50^b^: 40–10,700 nM Pearson’s correlation^a^: 0.7	Effective, universally applicable, require little input
35	NHGNN-DTA: a node-adaptive hybrid graph neural network for interpretable drug-target binding affinity prediction (He et al., 2023)	He et al.	Drug discovery/design	MSE, pearson’s coefficient, concordance index, mean reversion	MSE^b^: 0.196 CI: 0.914 mean reversion: 0.744	Universally applicable, robust, accurate
36	Synergizing attribute-guided latent space exploration (AGLSE) with classical molecular simulations to design potent pep-magnet peptide inhibitors to abrogate sars-cov-2 host cell entry (Ullah et al., 2025)	Ullah et al.	Drug discovery/design	RMSD, RMSF	RMSD^b^: 0.5–5.3 angstrom RMSF^b^: 2–9.3 angstrom	Cost saving, effective
37	Deep learning-based bioactive therapeutic peptide generation and screening (Zhang et al., 2023b)	Zhang et al.	Drug discovery/design	AUC, accuracy, TPR, precision, MCC	AUC^a^: 0.8673 TPR^a^: 0.8652 MCC^a^: 0.3239	Widely used, cost saving, quick Reliable, efficient
38	DMAMP: a deep-learning model for detecting antimicrobial peptides and their multi-activities (Meng et al., 2024)	Meng et al.	Drug discovery/design	MCC, F1 score, AUC	MCC^a^: 0.7298 F1 score^a^: 0.9273 AUC^a^: 0.9582	Quick, cost saving, efficient, effective
39	Active learning guided drug design lead optimization based on relative binding free energy modeling (Gusev et al., 2023)	Gusev et al.	Drug discovery/design	TI ΔΔG	TI ΔΔG: < 2.73 kcal/mol	Effective
40	Discovery of potential inhibitors of SARS-CoV-2 main protease by a transfer learning method (Zhang et al., 2023a)	Zhang et al.	Virtual Screening	AUROC, AUPRC, recall, accuracy, precision, f1 score, docking score	Docking score^b^: −0.85 to −10.4 AUROC^a^: 0.682 AUPRC^a^: 0.344 Recall^a^: 0.344 Accuracy^a^: 0.913 Precision^a^: 0.520 F1 score^a^: 0.413	Effective, quick
Computer aided drug design						
41	Deep-learning vs. physics-based docking tools for future coronavirus pandemics (Ma and Chen, 2025)	Ma and Chen	Drug discovery/design	RMSD	Accuracy^a^: 0.56 Å	Effective, quick
42	Multiview deep learning-based molecule design and structural optimization accelerates inhibitor discover (Pang et al., 2025)	Pang et al.	Drug discovery/design	docking score	Docking score^b^: 4.217	Quick
43	Designing novel inhibitor derivatives targeting SARS-CoV-2 Mpro enzyme: a deep learning and structure biology approach (Joshi et al., 2024)	Joshi et al.	Drug discovery/design	RMSD	RMSD^b^: 0.13–0.61	Quick, cost saving
44	In silico prediction of the inhibition of new molecules on SARS-CoV-2 3CL protease by using QSAR: PSOSVR approach (Madani et al., 2023)	Madani et al.	Drug discovery/design	RMSE, R^2, AIC	RMSE^b^: 0.1367 R^2^a^: 0.9665 AIC: − 249.1234	Effective, robust
45	A dual diffusion model enables 3D molecule generation and lead optimization based on target pockets (Huang et al., 2024)	Huang et al.	Drug discovery/design	affinity, QED, LogP	Affinity^a^: 0.628 QED: 0.594 LogP: 0.301	quick, effective, less complex
46	An approach combining deep learning and molecule docking for drug discovery of cathepsin L (Li et al., 2023)	Li et al.	Drug discovery/design	RMSD	RMSD^b^: 0.147A	Large scale application, quick, applicable for large dataset, decreased labour, cost saving
Generative adversarial networks						
47	Low-data drug design with few-shot generative domain adaptation (Liu J. et al., 2023)	Liu et al.	Drug discovery/design	QED, Plogp	QED: 0.771–0.769 Plogp: 0.672–0.682	Require little input, effective
48	AI-driven drug discovery: unravelling the potential of generative adversarial networks (GANs) in pharmaceutical research (Burri et al., 2023)	Burri et al.	Drug discovery/design	F1 score, accuracy, precision, recall, specificity, sensitivity, AUROC	F1-score^a^: 0.85, Recall^a^: 0.87, Accuracy^a^: 0.85, Precision^a^: 0.83, Specificity^a^: 0.82, Sensitivity^a^: 0.87, ROC-AUC^a^: 0.91	Quick, accurate, widely used, efficient
Deep neural network						
49	Artificial intelligence-aiding lab-on-a-chip workforce designed oral [3.1.0] bi and [4.2.0] tricyclic catalytic interceptors inhibiting multiple SARS-CoV-2 protomers assisted by double-shell deep learning (Kalasin and Surareungchai, 2024)	Kalasin and Surareungchai	Drug discovery/design	R^2, RMSE	R^2: 0.97 RMSE^b^ ranging: 0.20–0.40	Accurate, cost saving, large scale application, effective
50	MFE-ACVP: anti-coronavirus peptide prediction based on multimodal feature extraction and ensemble learning (Kang et al., 2025)	Kang et al.	Screening	accuracy, MCC, sensitivity, specificity, AUC	AUC^a^: 86.37% ACC^a^: 77.62% MCC^a^: 65.19% Sensitivity^a^: 71.61% Specificity^a^: 90.58%	Quick, cost saving, sensitive, universally applicable
51	Binding activity classification of anti-SARS-CoV-2 molecules using deep learning across multiple assays (Eren Yamasan and Korkmaz, 2024)	Eren Yamasan and Korkmaz	Binding affinity	BACC, MCC, AUC, F1 score, precision, recall	BACC^a^: 0.690–0.803 MCC^a^: 0.172–0.561 AUC^a^: 0.751–0.868 F1 score^a^: 0.135–0.0693 Precision^a^: 0.075–0.730 Recall^a^: 0.659–0.846	Quick, effective, applicable for large dataset
Graph neural networks						
52	Large-scale deep learning identifies the antiviral potential of PKI-179 and MTI-31 against coronaviruses (van der Horst et al., 2024)	van der Horst et al.	Drug discovery/design	IC50	IC50^b^: 100 to 50,000 nM	Quick, effective, universally applicable
53	Molecule generation toward target protein (SARS-CoV-2) using reinforcement learning-based graph neural network via knowledge graph (Ranjan et al., 2023)	Ranjan et al.	Drug discovery/design	CI, RMSE, MSE	CI: 0.902 RMSE^b^: 0.427 MSE^b^: 0.223	Effective
54	A knowledge-guided graph learning approach bridging phenotype- and target-based drug discovery (Ye et al., 2025)	Ye et al.	Drug discovery/design	RMSE, Pearson’s coefficient	RMSE improvement: 0.169 PCC improvement: 0.110	Effective, universally applicable, easy to interpret
55	AntiViralDL: computational antiviral drug repurposing using graph neural network and self-supervised learning (Zhang et al., 2023)	Zhang et al.	Drug Repurposing	AUC, AUPRC	Average AUC^a^: 0.8494	Effective
56	A graph neural network-based approach for predicting SARS-CoV-2–human protein interactions from multiview data (Ray et al., 2025)	Ray et al.	Computational predictions	ROC-AUC precision	ROC-AUC^a^: 83% Average precision score^a^: 82%	Universally applicable, robust, reliable
57	MGPPI: multiscale graph neural networks for explainable protein–protein interaction prediction (Zhao et al., 2024)	Zhao et al.	Computational predictions	accuracy, precision, recall, F1 score	Accuracy^a^: 82.53 Precision^a^: 81.77 Recall^a^: 83.68 F1 score^a^: 82.17	Effective, universally applicable

a The closer the value to 1/the higher the value, the better the metric performance (PCC, R^2, AUPRC, AUC, BACC, F1, specificity, sensitivity, precision, accuracy, QED, MCC).

b The lower the value, the better the metric performance (MAE, MSE, RMSE, RMSD, RMSF, docking score, binding energy, IC50, EC50).

In Table 4, almost all the included literature reviewed were cases applied in the pre-commercialisation stage, where the final product is yet to be launched into the market. For instance, in the area of drug repurposing, instead of starting on a completely new compound, researchers worked on an existing drug candidate (Zhang et al., 2023). The most common technology was Deep Learning (35.1%) followed by AI/ML and virtual screening (14.0% each) while the most common research area was drug discovery/design (54.4%) followed by computational prediction (28.1%).

We also identified keywords used in the conclusion of articles describing the technology model implemented, usually in comparison with another similar or older model. A coding manual (Appendix E) was created to establish these keywords. As shown in Table 4, “Effective” is the most common keyword (66.7%), with performance metrics such as EC50 values presented. 36.8% of the articles used “Quick” or “Time saving” and proved it by presenting AUCROC values. 24.6% of articles used “Accurate” to describe their model, substantiated by accuracy percentages. Other articles used other terms like “Cost Saving” and “Widely Applicable.” As each piece of literature may mention more than one keyword, the total percentage of the keywords may not total to 100%. Most of the conclusions made from the articles are relating to the strengths of the models (Research Objective 2) with little to no information made about Research Objectives 1 and 3. This might be due to the infancy of AI in the pharmaceutical workflow where research is mainly focused on the early stages of predictive discovery.

## 4 Discussion

In this study, the included articles highlighted the nonclinical performance metrics in different settings of the early phase of pharmaceutical development. However, they offer limited insight into how these technological developments translate into clinically meaningful pharmacological effects in the investigated compounds. The laborious steps requiring high-throughput screening and optimisation in early product development pose a challenge for pharmaceutical companies. This underscores the need for a reliable technological system to speed up and aid repetitive processes, while facilitating the decision-making process. This is further discussed in the proceeding sections.

### 4.1 Predominance of AI predictive metrics over experimental metrics

From Figure 4, it is observed that AI/ML model evaluation metrics, such as the regression metrics and classification and curve metrics, are reported the most frequently in the methodologies and results section of articles. The apparent predominance of AI evaluation metrics over drug evaluation and computational evaluation combined shows the emphasis of AI research algorithm benchmarking. The research gap that most articles (Ma and Lei, 2024; Wang et al., 2023; Ren et al., 2023) are geared towards developing and comparing the AI and statistical models. The AI/ML model evaluation metrics allow researchers to compare and rank these models’ performances with greater precision and accuracy, attesting to its widespread adoption. This showcases the emphasis on algorithmic performance in early-stage research.

In addition, most of the AI/ML model evaluation metrics reported are classification and curve metrics, such as accuracy and precision, as compared to fewer mentions of regression metrics such as MSE and R (Yin et al., 2017). This indicates that many predictive models are designed for categorical prediction tasks, such as identifying active compounds. These classification tasks are commonly associated with research activities in the early stages of the drug-development pipeline. In these fields, AI predictive models are tasked to identify a small set of potentially viable candidates from a large batch of molecules, making classification and curve metrics a more suitable indicator. Consequently, the prevalence of these metrics further highlights the emphasis on upstream processes rather than downstream pharmacological evaluation.

These observations suggest that AI technologies are currently most widely applied in the early discovery stages of the pharmaceutical pipeline. In the post-pandemic context, the high mortality and rapid spread of COVID-19 created an urgent need to accelerate upstream processes and shorten the drug development pipeline. The surge of COVID-19 drug development and repurposing studies are heavily centred on discrete classification of potential lead compounds. The screening of large chemical libraries to identify a small subset of active compounds often used classification and curve-based metrics (AUROC, AUPRC), as they measure the model’s ability to discriminate between classes across different decision thresholds, unlike regression metrics. Thus, the gradual improvements of AI predictive models can accelerate and streamline the research stage of drug development, where rapid computational prioritisation of compounds is most valued.

However, the low representation of pharmacologically meaningful drug candidate evaluation and computational evaluation highlights a potential gap between algorithmic performance and practical pharmaceutical outcomes. Many research studies are centred around assessing and evaluating the predictive model, while studies on the pharmacological effects of unique compounds are limited (Wu et al., 2025; Qian et al., 2025). The incidence of drug candidate evaluation metrics is mainly from a small range of studies investigating the activity of molecular substitution of specific compounds or for specific targets (Li Z. et al., 2025; Gusev et al., 2023). This may suggest a possible mismatch between reported performance metrics and the end goal of drug development. The positive performance of a machine learning model may not directly guarantee positive therapeutic outcomes of the investigated compound. As these articles are technology centred rather than clinically centred, they cannot be solely utilised to conclude that the pharmaceutical products being researched on are clinically beneficial.

Subsequent transition to greater predictive model training surrounding compounds’ effects on the body, such as pharmacokinetics and pharmacodynamics, could broaden the scope of future research. Such integration would help bridge the gap between algorithmic performance and clinical outcomes, where AI can better contribute to turning drug discovery efforts into effective pharmaceutical therapies.

### 4.2 Applications of Pharma 4.0 technology and performance metrics in research areas

As depicted in Table 4, distinct technologies and performance metrics demonstrate varying degrees of prevalence within specific areas. This section critically examines these patterns, highlighting how such tools are employed and optimized within each research context. These patterns provide insight into how early-stage pharmaceutical development is heavily directed towards computational efficiency and optimisation instead of clinical translation.

#### 4.2.1 Applications of Pharma 4.0 technology in research areas

Machine learning models, particularly those incorporating virtual screening, computer-aided drug design (CADD), generative adversarial networks (GANs), and graph neural networks (GNNs), were predominantly applied in drug discovery and drug design (Eugster et al., 2024; Gantla et al., 2023; Li Z. et al., 2025; Ma and Lei, 2024; Ma and Chen, 2025; Liu W. et al., 2023; Burri et al., 2023; van der Horst et al., 2024; Ranjan et al., 2023). The nature of these models is particularly suitable for generating and optimising molecular structures necessary in discovering new drugs. Hence, these Machine Learning models are used to predict biological activity and pharmacological properties effectively, addressing the challenges associated with labour intensive high-throughput screening mentioned earlier.

Building on these Machine Learning applications, Deep Learning emerged as another dominant technology in the areas of drug discovery, drug design (Yang et al., 2025; Zhang et al., 2024; Ma and Lei, 2024), and computational prediction (Reilly et al., 2025; Zhang et al., 2025; Ren et al., 2023). Compared with conventional Machine Learning methods, Deep Learning models are capable of learning complex, nonlinear relationships directly from large-scale molecular and biological datasets with minimal manual feature engineering. This capability enables more accurate prediction of biological activities, pharmacological properties, and other computational endpoints while capturing biological patterns that are often difficult to model using traditional machine learning methods. Consequently, Deep Learning serves as a complementary computational tool that extends the predictive capabilities established by broader Machine Learning techniques, further supporting data-driven decision-making throughout the drug discovery pipeline.

While Deep Learning offers advantages in modelling highly complex datasets, more traditional machine learning algorithms such as the Random Forest (RF) model is particularly utilised in computational prediction tasks (Elia and Duan, 2025). It is valued for its strong predictive accuracy, robustness against overfitting, and ability to capture nonlinear relationships which makes it particularly suitable for regression and classification problems. In addition, their relatively high interpretability compared with many deep learning models enhances confidence in predicting pharmacological endpoints, including biological activity and toxicity. As a result, Random Forest continues to provide a reliable and practical alternative for computational prediction, complementing more sophisticated deep learning methods.

In contrast to the patterns observed for Machine Learning, Deep Learning, and Random Forest models, no distinct trends were identified for technologies such as support vector machines and deep neural networks. This is likely attributable to the relatively small number of studies involving these technologies within the scope of this review, limiting the ability to identify consistent application domains. Nevertheless, their inclusion demonstrates the diversity of computational approaches currently being explored in pharmaceutical research and highlights the continuing evolution of AI driven methodologies in drug discovery.

Overall, these findings reflect how different technology models are utilised in their respective fields according to their strengths, weaknesses and demands of each domain. In the context of early-phase pharmaceutical development, where processes such as high-throughput screening and optimisation remain labour-intensive and time-consuming, Pharma 4.0 technologies can be a critical solution to tackle these issues. By leveraging machine learning and computational tools to automate repetitive tasks, enhance predictive accuracy, and support data-driven decision-making, these technologies mitigate the inefficiencies highlighted earlier. However, consistent with the initial observation of this discussion, their application remains largely confined to improving nonclinical efficiency, with limited direct linkage to clinically meaningful pharmacological outcomes.

#### 4.2.2 Applications of performance metrics in research areas

Drug discovery and drug design studies most frequently employed IC50, AUC, F1 score, RMSE, and accuracy (Boby et al., 2023; Ren et al., 2023; Djeddi et al., 2023; Madani et al., 2023) as performance metrics. These studies commonly involve classifying lead compounds and predicting molecular properties, requiring a combination of different performance metrics. Metrics such as F1 score, AUC, and accuracy evaluate the model’s ability to quantify drug activity, while RMSE is often used to assess the accuracy of predicted IC50 values, which reflect drug potency. This combination of metrics integrates biological understanding with computational prediction to improve candidate prioritisation during early drug discovery. However, despite enhancing screening efficiency, these metrics primarily quantify predictive performance rather than clinical efficacy, meaning that compounds identified as promising computationally may not necessarily demonstrate meaningful therapeutic outcomes.

Building upon these predictive tasks, computational prediction studies similarly relied on AUC, accuracy, RMSE, and precision (Zhang et al., 2024; Ma and Lei, 2024; Wang et al., 2023) to evaluate machine learning model performance. These studies are primarily concerned with predicting biological activity, binding affinity, or other pharmacological properties, with emphasis placed on the accuracy and reliability of computational algorithms. Although these metrics provide robust measures of algorithmic performance, they remain focused on computational validation and do not directly indicate whether the predicted compounds will translate into clinically relevant therapeutic benefits.

Many computational prediction studies further extend into docking studies, where R^2^ and RMSE (Zamitalo et al., 2023; Shi et al., 2023) were the most commonly reported metrics, reflecting the regression-based nature of predicting binding affinity values and docking scores. R^2^ measures how well predicted values explain observed variation, while RMSE quantifies prediction error, together providing an indication of model reliability in estimating ligand–target interactions. Nevertheless, although these metrics offer quantitative evidence of molecular interactions, they do not adequately capture the complexity of downstream pharmacological responses or clinical effectiveness, highlighting another limitation of relying solely on computational evaluation.

The outcomes generated through docking studies often feed directly into virtual screening, where large libraries of compounds are prioritised for further investigation. In this domain, AUCROC (Zhang et al., 2023a) was the dominant evaluation metric because it measures how effectively a screening method ranks active compounds ahead of inactive ones across varying classification thresholds. This is particularly valuable in addressing the labour-intensive nature of high-throughput screening, enabling rapid identification of promising candidates from extensive molecular datasets. However, it provides limited information regarding downstream biological validation, reinforcing the disconnect between computational efficiency and eventual clinical applicability.

Similarly, drug repurposing studies predominantly utilised AUC (Zhang et al., 2023) to evaluate the performance of models predicting drug–disease associations. Since these datasets are frequently imbalanced, with relatively few positive drug candidates among many negatives, AUC offers a robust assessment of ranking performance independent of classification thresholds, thereby streamlining the candidate selection process. Despite these advantages, the metric remains centred on computational discrimination rather than therapeutic validation, meaning that highly ranked repurposed drugs still require substantial experimental and clinical investigation before demonstrating real-world efficacy.

In contrast to the research areas discussed above, no dominant evaluation metric was identified for studies focusing on structure–activity relationships and binding affinity. This is again, likely attributable to the relatively small number of studies included in this review, preventing consistent trends from being established.

Overall, while these performance metrics effectively evaluate nonclinical performance, they remain largely concentrated on computational and early-stage validation. This reinforces the observation that current approaches in early pharmaceutical development are heavily focused on optimising technical performance metrics, with limited translation to clinically meaningful pharmacological outcomes. Despite their utility in streamlining laborious processes like high-throughput screening and compound prioritisation, these metric-driven evaluations highlight an ongoing gap between computational efficiency and real-world clinical implications.

### 4.3 Implications of Pharma 4.0 in pharmaceutical development

Based on the results and above analysis, it can be deduced that the implementation of Pharma 4.0 technology brings about numerous benefits to research processes in the early-stage product life cycle. It aids in the streamlining of processes and facilitates overall productivity, enabling better and faster results (Ang et al., 2023; Pal et al., 2025; Ye et al., 2025).

Though these benefits are primarily concentrated in the nonclinical domain as mentioned earlier, the versatility and scalability of these technologies highlight their broader potential. The wide applicability of various Pharma 4.0 technologies across different fields allows for more diverse disease targeting and cheaper production since the same methods could possibly be used for different purposes (Zhang et al., 2023b; Li et al., 2023; Zhang et al., 2023). This could mean that in the event of another pandemic similar to COVID-19, Pharma 4.0 technologies could be implemented to readily repurpose or discover new cures within short periods of time.

As such, we believe that Pharma 4.0 in pharmaceutical companies display massive capabilities to raise industrial standards to greater heights. The inevitable proliferation of Pharma 4.0 technologies brings about heavy downstream improvements, with their benefits reaching the healthcare system. These technologies make companies more resilient in navigating through health emergencies while advancing drug development and improving clinical outcomes. However, to fully realise its potential, more must be done to move beyond optimising computational performance and instead focus more on translating them to meaningful clinical outcomes.

### 4.4 Limitations and suggestions for future studies

Our study considered only two databases (Scopus and PubMed) due to time and resource constraints. The articles included in this study were also restricted to those published between 2023 and 2025.

In our consideration, the two databases chosen are the largest multidisciplinary biomedical databases of peer-reviewed journals to search and cover relevant published studies. As we are focusing on the applicability of Pharma 4.0 technologies after the pandemic, 2023 to 2025 was determined as the optimal period to obtain the most updated published articles. While we acknowledge a narrower scope and smaller sample size of articles could have led to omission of other relevant studies in other databases or earlier years, this study is still useful to provide a preliminary finding on the current insights and the changes made by the contemporary Pharma 4.0 in pharmaceutical development in the post-pandemic period.

As with all published articles, publication bias may exist. The nature of studies available in this field may lead to the tendency for only positive results to be reported, which may overestimate the practical benefits of Pharma 4.0 technologies. This publication bias could influence the interpretation of the article. The high proportion of studies of high risk of bias should also be considered in relation to potential publication bias. Consequently, favorable outcomes reported by studies of high risk of bias should be interpreted cautiously, as the overall evidence base may be influenced by both methodological limitations and selective publication. Our study approach to adopt a systematic review hopes to mitigate these risks. Our future study could consider a longer time period to enhance the reliability of the evidence and analysis.

Another limitation of this review is that no prospective review protocol was registered. Although the research questions, eligibility criteria, search strategy, study-selection process, and data-extraction procedures were predefined, prospective protocol registration would have further enhanced the transparency and reproducibility of the review and reduced the potential for *post hoc* methodological decisions. Future reviews in this rapidly evolving field should consider prospective protocol registration and broader database coverage, including appropriate consideration of preprint and grey literature sources.

### 4.5 Conclusion

The contemporary AI-enabled Pharma 4.0 applications and predictive modelling show benefits in pharmaceutical drug development by optimising stages in the product life cycle, particularly in early drug discovery. Our systematic review suggested that many of these Pharma 4.0 applications reported favourable predictive performance, and could be useful for quantification of drug product properties. This systematic review identified diverse applications of AI/ML predictive models across processes including drug candidate evaluation, computational modelling, virtual screening, and related discovery activities. Many included models reported favourable predictive performance based on the AI/ML evaluation metrics used within individual studies, suggesting that these models may provide tangible benefits such as shorter lead time and greater productivity. However, clinical effectiveness and patient outcomes were not established by the included evidence. Further externally validated and prospective studies are required to determine whether these advantages translate into reduced development time and costs or improved clinical outcomes. In this regard, the findings should be interpreted cautiously because most included studies were assessed as having high risk of bias and lacked external validation. Overall, the current evidence supports the potential utility of AI-enabled Pharma 4.0 applications and predictive modelling as complementary tools in early-stage drug discovery, rather than establishing lifecycle-wide or clinical benefits.

## Data Availability

The datasets presented in this study can be found in online repositories. The names of the repository/repositories and accession number(s) can be found in the article/Supplementary material.
