# Supplementary material for: Navigating the Pharma 4.0 revolution: how AI-enabled applications and predictive modelling shaped pharmaceutical development in the post-pandemic era: a systematic review
Source: Front Artif Intell. 2026 Oct 30;9:1976101. doi: 10.3389/frai.2026.1976101 (PMC13760604; doi:10.3389/frai.2026.1976101)
Supplement: Supplementary file 1 [file Data_Sheet_1.pdf]

Appendix

Appendix A

List of Pharma 4.0 Technologies	
Artificial Intelligence (AI)	Application of computer systems capable of performing tasks that require human intelligence, especially by applying machine learning techniques to massive amounts of data
Machine Learning (ML)	Algorithms that learn and adapt to patterns from datasets to make predictions or classifications without explicit programming
Virtual Screening	Computational technique to scan massive chemical libraries to identify the most promising candidate that binds to a specific biological target
Random Forest Model	A classical machine learning algorithm that combines the output of multiple decision trees to reach a single result
Deep Learning (DL)	A type of machine learning that use multi-layer neural networks to learn complex patterns from large dataset
Computer Aided Drug Design (CADD)	Computational techniques used to design and evaluate potential drug candidates
Support Vector Machines (SVM)	A supervised machine learning algorithm primarily used for classification and regression tasks
Generative Adversarial Networks (GAN)	Machine learning frameworks designed to create new, synthetic data instances that resemble a given training dataset
Deep Neural Networks (DNN)	Neural networks with multiple hidden layers that enable high-dimensional feature learning
Generative Neural Networks (GNN)	Deep learning models designed for graph-structured data
Cloud Computing	The on-demand access to shared computing resources, including servers, storage, databases and software over internet networks
Internet of Things	Networks of interconnected physical divides embedded with sensors, software and processing ability, enabling them to collect, exchange and analyse data in real time.
Robotics and Automation	The use of technology, software or machinery to perform tasks with minimal human interaction.
Big Data	Extremely large and complex dataset that require high computational and processing ability

Appendix B

List of Performance Metrics	
pLogP	Measures lipophilicity of molecules
RMSD (Root Mean Square Deviation)	Measures the structural similarity between predicted and actual molecular conformation
RMSF (Root Mean Square Fluctuation)	Calculation used in molecular dynamics simulation to measure the flexibility or mobility of individual residues or atoms
Docking score	A quantitative value from a computational algorithm predicting the binding affinity between ligand and protein
Binding energy	The minimum energy required to dissemble a bound system, in particular ligand-protein interaction
EC50	The concentration of a drug required to achieve 50% of its maximal effect
IC50/pIC50	The concentration of a substance required to inhibit a biological process by 50%
AUC/AUROC/AUC -ROC	Measures the ability of the model to distinguish between classes of classification (true/false, positive/negative) cross different thresholds
AUPRC (Area Under Precision Recall Curve)	Measures the trade-off between precision and recall across different classification thresholds
MCC (Matthews correlation coefficient)	A balanced performance metric for classification models in machine learning that account for class imbalance
Accuracy (ACC)	Proportion of correctly predicted instances among all predictions, calculated as the ratio of correct predictions to the total number of predictions
Precision	Proportion of predicted positive results that are truly positive, calculated as the ratio of true positives to all predicted positives
Sensitivity/Recall /True Positive Rate (TPR)	Ability of the model to correctly identity true positive cases, calculated as the ratio of true positives to true positives plus false negatives
Specificity	Ability of the model to correctly identify true negatives, calculated as a ratio of true negatives to true negatives over false positives
BACC (Balanced accuracy)	The average of sensitivity and specificity

F1 score	Machines learning evaluation metric that represents the harmonic mean of precision and recall, balancing false positives and false negatives
Root Mean Square Error (RMSE)	Measures the average magnitude of prediction error
MSE (Mean Square Error)	Measures the average squared difference between predicted and actual values
MAE (Mean Absolute Error)	Measures the absolute difference between predicted and actual values
R^2 (Coefficient of determination)	Measures the proportion of variance in the dependent variable explained by independent variables
Pearson's coefficient (R)	Measures the linear relationship between predicted and actual values

Appendix C

**List of Research Areas**	
Drug discovery/design	To identify new therapeutic compounds and design new molecules that are able to treat diseases
Drug repurposing	Finding new purpose for already existing drugs/compounds
Computational prediction	Using computer-based models to predict biological, chemical properties or clinical outcomes.
Virtual screening	Identification of potential drug candidates to be used in drug discovery/design
Docking studies	Analysis of docking results of molecules to evaluate their binding ability
Binding affinity	Measure of how strongly a molecule binds to its target
Structured Activity Relationship (SAR)	Study of how a change in the structure of the molecule would affect its biological activity

Appendix D

Full Search Terms	
**Pubmed**	SARS-CoV-2[Mesh] OR "COVID-19"[Mesh] OR "Coronavirus Infections"[MeSH Terms] OR 2019-nCoV[Title/Abstract] OR 2019 ncov[Title/Abstract] OR nCov[Title/Abstract] OR covid-19[Title/Abstract] OR covid19[Title/Abstract] OR COVID-2019[Title/Abstract] OR COVID2019[Title/Abstract] OR sars-cov-2[Title/Abstract] OR "sars cov-2"[Title/Abstract] OR sarscov2[Title/Abstract] OR sarscov-2[Title/Abstract] OR sars-coronavirus-2[Title/Abstract] OR sars corona virus[Title/Abstract] OR sars-like coronavirus[Title/Abstract] OR novel coronavirus[Title/Abstract] OR novel corona virus[Title/Abstract] OR covid*[Title/Abstract] OR coronavirus 2[Title/Abstract] OR coronavirus infection*[Title/Abstract] OR coronavirus disease[Title/Abstract] OR corona virus disease[Title/Abstract] OR new coronavirus[Title/Abstract] OR new corona virus[Title/Abstract] OR new coronaviruses[Title/Abstract] OR novel coronaviruses[Title/Abstract] OR severe acute respiratory syndrome coronavirus-2[Title/Abstract] OR ((coronavirus[Title/Abstract] OR ncov[Title/Abstract] OR sars-cov[Title/Abstract]) AND (2019[Title/Abstract] OR 19[Title/Abstract] OR Wuhan[Title/Abstract])) AND<br><br>("Pharma 4.0"[Title/Abstract] OR "Industry 4.0"[Title/Abstract] OR "artificial intelligence"[MeSH Terms] OR "artificial intelligence"[Title/Abstract] OR "machine learning"[MeSH Terms] OR "machine learning" [Title/Abstract] OR "automation" [MeSH Terms] OR "automation"[Title/Abstract] OR "cloud computing"[MeSH Terms] OR "cloud computing"[Title/Abstract] OR "digital transformation"[Title/Abstract] OR "smart manufacturing"[Title/Abstract] OR “internet of things” [Title/Abstract] OR “IoT” [Title/Abstract]) AND<br><br>(pharmaceutical research[MeSH Terms] OR drug development*[MeSH terms] OR pharmaceutical development [Title/Abstract] OR medication development [Title/Abstract] OR pharmaceutical manufacturing [Title/Abstract] OR drug manufacturing [Title/Abstract] OR pharmaceutical polic* [Title/Abstract] OR drug regulation [Title/Abstract] OR drug quality control [Title/Abstract] OR (drug discovery*[MeSH terms])<br><br>+ Published date filter: 2023-2025
**Scopus**	("covid-19" or covid19 or nCov or "sars-cov-2" or sarscov2 or "sarscov-2" or "sars-coronavirus-2" or "sars corona virus" or "sars-like coronavirus" or "novel coronavirus" or "novel corona virus" or covid* or "coronavirus 2" or "coronavirus infection*" or "coronavirus disease" or "corona virus disease" or "new coronavirus" or "new corona virus" or "new coronaviruses" or "novel coronaviruses" or "severe acute respiratory syndrome coronavirus-2") OR ((coronavirus OR ncov OR sars-cov) W/5 (2019 OR 19 OR Wuhan)) AND<br><br>"Pharma 4.0" OR "Industry 4.0" OR "artificial intelligence" OR "machine learning" OR "automation" OR "cloud computing" OR "digital transformation" OR "smart manufacturing" OR “internet of things” OR “IoT” AND<br><br>"pharmaceutical research" OR "drug development*" OR "pharmaceutical development"OR "medication development"OR "drug development*" OR "pharmaceutical manufacturing" OR "drug manufacturing" OR "pharmaceutical polic*" OR "drug regulation" OR "drug quality control" OR "drug discovery*"<br><br>+ Published date filter: 2023-2025

Appendix E

Coding manual	
**Terms mentioned in article → Key words used in conclusion**	
“Highly accurate”<br>“Able to effectively predict”<br>“Outperformed”<br>“Achieves intended results”	Effective
“High accuracy”<br>“Less error variability”<br>“Low error rates”<br>“Precise”	Accurate
“Cutting down time required”<br>“Promoting rapid screening”<br>“Facilitating decision making”<br>“Accelerating”<br>“Less time consuming”<br>“Expedite”<br>“Fast”	Quick
“Can extend predictions to other areas”<br>“Highly generalisable”<br>“Inherent extensibility”<br>“Scalable”<br>“Transferable”<br>“Broadly applicable”	Universally applicable
“Consistent”<br>“Reproducible”	Reliable
“Demonstrates high robustness”<br>“Performs consistently under varying conditions”	Robust
“Suitable for large datasets”<br>“Scalable to large data sets”<br>“Capable of handling large datasets”	Applicable for large dataset
“Cutting down the cost required”<br>“Computational costs significantly lower”<br>“Cheaper”	Cost saving
“Save time and effort”<br>“Efficient”<br>“Streamlines processes”	Efficient
“Widely adopted”<br>“Extensively used”<br>“Adopted in numerous fields”	Widely used
“Requires minimal input”	Require little input

“Less complexity”<br>“More straightforward”<br>“Simpler”<br>“Less complicated”	Less complex
“Targeted”<br>“Precise”<br>“Highly specific”	Specific
“Highly sensitive”<br>“High detection capability”	Sensitive
“Reduced workload”<br>“Less labour intensive”<br>“Lower labour requirements”	Decreased labour
“Increases interpretability”<br>“Straightforward to interpret”<br>“Readily interpretable”	Easy to interpret

Appendix F

No.	Title	Author	Research Area	Risk of Bias#
**Artificial Intelligence/ Machine Learning**				
1	Open science discovery of potent noncovalent SARS-CoV-2 main protease inhibitors[13]	Melissa L Boby et. al	Drug discovery/ design	Unclear
2	AI-accelerated protein-ligand docking for SARS-CoV-2 is 100-fold faster with no significant change in detection[15]	Clyde, Austin et. al	Docking studies	Unclear
3	Artificial intelligence-based HDX (AI-HDX) prediction reveals fundamental characteristics to protein dynamics: Mechanisms on SARS-CoV-2 immune escape[16]	Jiali Yu et. al	Computational Prediction	Unclear
4	Leveraging machine learning to streamline the development of liposomal drug delivery systems[17]	Remo Eugster et. al	Computational Prediction	Unclear
5	Repurposing of drugs for combined treatment of COVID-19 cytokine storm using machine learning[18]	Maanaskumar R Gantla	Computational Prediction	High
6	FragOPT: An ML-Driven Computational Workflow for Rational Fragments Optimization Toward Lead Compounds[19]	Xiaoyan Wu et. al	Drug discovery/ design	Unclear
7	De Novo Design of Anti-COVID Drugs Using Machine Learning-Based Equivariant Diffusion Model Targeting the Spike Protein[20]	Vidya Niranjan et. al	Drug discovery/ design	Unclear
8	Accelerating reliable multiscale quantum refinement of protein–drug systems enabled by machine learning[21]	Zeyin Yan et. al	Drug discovery/ design	Unclear
**Virtual Screening**				
9	Virtual and In Vitro Screening of Natural Products Identifies Indole and Benzene Derivatives	Dony Ang et. al	Virtual Screening	Unclear

No.	Title	Author	Research Area	Risk of Bias#
	as Inhibitors of SARS-CoV-2 Main Protease (Mpro)[22]			
10	Accelerating Molecular Docking using Machine Learning Methods[23]	Abdulsalam Y Bande et. al	Docking studies	Unclear
11	Development of Machine Learning Regression Model for COVID-19 Drug Target Prediction[24]	Alexandra Zamitalo et. al	Docking studies	High
12	Effective Surrogate Models for Docking Scores Prediction of Candidate Drug Molecules on SARS-CoV-2 Protein Targets[25]	Wenqi Shi et. al	Docking studies	High
13	Artificial Intelligence-Driven Discovery of Pyrazolo[1,5-a]pyrimidine Derivatives as Novel Phosphodiesterase 4 Inhibitors for Treating Idiopathic Pulmonary Fibrosis[26]	Zheng Li et. al	Drug discovery/ design	Unclear
14	Applications of Machine Learning Approaches for the Discovery of SARS-CoV-2 PLpro Inhibitors[27]	Sourav Pal et. al	Drug discovery/ design	Unclear
15	Modeling structure–activity relationships with machine learning to identify GSK3-targeted small molecules as potential COVID-19 therapeutics[28]	Rameez Hassan Pirzada et. al	Drug discovery/ design	Unclear
16	D2Screen: Embedding Pretrained Representation Learning Model and Molecular Docking for Virtual Screening[29]	Tingli Qian et. al	Drug discovery/ design	Unclear
		**Random Forest Model**		
17	Machine Learning Approaches for COVID-19 Classification and Potential Novel Therapeutic Targets[31]	Elia, Marina G. et. al	Computational Prediction	High
18	Developing a SARS-CoV-2 main protease binding prediction random forest	Jie Liu et. al	Computational Prediction	Unclear

No.	Title	Author	Research Area	Risk of Bias#
	model for drug repurposing for COVID-19 treatment[32]			
	**Support Vector Machine**			
19	Proteomic data and structure analysis combined reveal interplay of structural rigidity and flexibility on selectivity of cysteine cathepsins[33]	Livija Tušar et al.	SAR	Unclear
20	Quantitative Structure–Activity Relationship Study of Cathepsin L Inhibitors as SARS-CoV-2 Therapeutics Using Enhanced SVR with Multiple Kernel Function and PSO[34]	Shaokang Li et al.	Drug discovery/ design	Unclear
	**Deep Learning**			
21	Integrating Physics-Based Simulations with Data-Driven Deep Learning Represents a Robust Strategy for Developing Inhibitors Targeting the Main Protease[35]	Yanqing Yang et. al	Computational Prediction	Unclear
22	F-CPI: A Multimodal Deep Learning Approach for Predicting Compound Bioactivity Changes Induced by Fluorine Substitution[36]	Qian Zhang et. al	Computational Prediction	Unclear
23	A deep learning framework for predicting molecular property based on multi-type features fusion[37]	Mei Ma et. al	Computational Prediction	High
24	A Robust Drug–Target Interaction Prediction Framework with Capsule Network and Transfer Learning[38]	Yixian Huang et. al	Computational Prediction	Unclear
25	How Deep Learning in Antiviral Molecular Profiling Identified Anti-SARS-CoV-2 Inhibitors[39]	Mohammed Ali et. al	Computational Prediction	High
26	AMMVF-DTI: A Novel Model Predicting Drug–Target Interactions	Lu Wang et. al	Computational Prediction	High

No.	Title	Author	Research Area	Risk of Bias#
	Based on Attention Mechanism and Multi-View Fusion[40]			
27	Protein-ligand binding affinity prediction using multi-instance learning with docking structures[41]	Hyojin Kim et. al	Computational Prediction	High
28	Chem2Side: A Deep Learning Model with Ensemble Augmentation (Conventional + Pix2Pix) for COVID-19 Drug Side-Effects Prediction from Chemical Images[43]	Arshed, Muhammad Asad et. al	Computational Prediction	High
29	Improving Protein–Ligand Interaction Modeling with cryo-EM Data, Templates, and Deep Learning in 2021 Ligand Model Challenge[44]	Nabin Giri et. al	Computational prediction	Unclear
30	Broad-spectrum coronavirus inhibitors discovered by modeling viral fusion dynamics[45]	Charles B Reilly et. al	Drug discovery/ design	Unclear
31	MVCL-DTI: Predicting Drug-Target Interactions Using a Multiview Contrastive Learning Model on a Heterogeneous Graph[46]	Bei Zhang et. al	Drug discovery/ design	Unclear
32	DeepMPF: deep learning framework for predicting drug-target interactions based on multi-modal representation with meta-path semantic analysis[47]	Zhong-Hao Ren et. al	Drug discovery/ design	High
33	Advancing drug–target interaction prediction: a comprehensive graph-based approach integrating knowledge graph embedding and ProtBert pretraining[48]	Warith Eddine Djeddi et. al	Drug discovery/ design	High
34	Unsupervised deep learning for molecular dynamics simulations: a novel analysis of	Jessica Mustali et. al	Drug discovery/ design	High

No.	Title	Author	Research Area	Risk of Bias#
	protein-ligand interactions in SARS-CoV-2 Mpro[49]			
35	NHGNN-DTA: a node-adaptive hybrid graph neural network for interpretable drug-target binding affinity prediction[50]	Haohuai He et. al	Drug discovery/ design	Unclear
36	Synergizing Attribute-Guided Latent Space Exploration (AGLSE) with Classical Molecular Simulations to Design Potent Pep-Magnet Peptide Inhibitors to Abrogate SARS-CoV-2 Host Cell Entry[51]	Farhan Ullah et. al	Drug discovery/ design	High
37	Deep Learning-Based Bioactive Therapeutic Peptide Generation and Screening[52]	Haiping Zhang et. al	Drug discovery/ design	High
38	DMAMP: A Deep-Learning Model for Detecting Antimicrobial Peptides and Their Multi-Activities[53]	Qiaozhen Meng et.al	Drug discovery/ design	High
39	Active Learning Guided Drug Design Lead Optimization Based on Relative Binding Free Energy Modeling[54]	Filipp Gusev et. al	Drug discovery/ design	High
40	Discovery of Potential Inhibitors of SARS-CoV-2 Main Protease by a Transfer Learning Method[55]	Huijun Zhang	Virtual Screening	High
	**Computer Aided Drug Design**			
41	Deep-Learning vs Physics-Based Docking Tools for Future Coronavirus Pandemics[57]	Yizhou Ma et. al	Drug discovery/ design	High
42	Multiview Deep Learning-Based Molecule Design and Structural Optimization Accelerates Inhibitor Discover[58]	Chao Pang et. al	Drug discovery/ design	High
43	Designing novel inhibitor derivatives targeting SARS-CoV-2 Mpro enzyme: a deep learning	Joshi, T. et. al	Drug discovery/ design	High

No.	Title	Author	Research Area	Risk of Bias#
	and structure biology approach[59]			
44	In silico prediction of the inhibition of new molecules on SARS-CoV-2 3CL protease by using QSAR: PSOSVR approach[60]	Achouak Madani et. al	Drug discovery/ design	High
45	A dual diffusion model enables 3D molecule generation and lead optimization based on target pockets[61]	Lei Huang et. al	Drug discovery/ design	High
46	An approach combining deep learning and molecule docking for drug discovery of cathepsin L[62]	Qi Li et. al	Drug discovery/ design	High
	**Generative Adversarial Networks**			
47	Low-Data Drug Design with Few-Shot Generative Domain Adaptation[63]	Liu et al	Drug discovery/ design	Unclear
48	AI-Driven Drug Discovery: Unravelling the Potential of Generative Adversarial Networks (GANs) in Pharmaceutical Research[64]	Srinivasa et al	Drug discovery/ design	High
	**Deep Neural Network**			
49	Artificial intelligence-aiding lab-on-a-chip workforce designed oral [3.1.0] bi and [4.2.0] tricyclic catalytic interceptors inhibiting multiple SARS-CoV-2 protomers assisted by double-shell deep learning[65]	Kalasin, Surachate et. al	Drug discovery/ design	Unclear
50	MFE-ACVP: anti-coronavirus peptide prediction based on multimodal feature extraction and ensemble learning[66]	Liqiong Kang et. al	Screening	High
51	Binding Activity Classification of Anti-SARS-CoV-2 Molecules using Deep Learning Across Multiple Assays[67]	Bilge Eren Yamasan et. al	Binding affinity	High
	**Graph Neural Networks**			

No.	Title	Author	Research Area	Risk of Bias#
52	Large-scale deep learning identifies the antiviral potential of PKI-179 and MTI-31 against coronaviruses[68]	Demi van der Horst et. al	Drug discovery/ design	Unclear
53	Molecule generation toward target protein (SARS-CoV-2) using reinforcement learning-based graph neural network via knowledge graph[69]	Amit Ranjan et. al	Drug discovery/ design	High
54	A Knowledge-Guided Graph Learning Approach Bridging Phenotype- and Target-Based Drug Discovery[70]	Qing Ye et. al	Drug discovery/ design	High
55	AntiViralDL: Computational Antiviral Drug Repurposing Using Graph Neural Network and Self-Supervised Learning[71]	Pan Zhang et. al	Drug Repurposing	Unclear
56	A graph neural network-based approach for predicting SARS-CoV-2–human protein interactions from multiview data[72]	Sumanta Ray et al	Computational predictions	Unclear
57	MGPPI: multiscale graph neural networks for explainable protein–protein interaction prediction[73]	Shiwei Zhao et. al	Computational predictions	Unclear

#This is derived based using the PROBAST form
